# Supplementary material for: A miniature dialysis-culture device allows high-density human-induced pluripotent stem cells expansion from growth factor accumulation
Source: Commun Biol. 2021 Nov 19;4:1316. doi: 10.1038/s42003-021-02848-x (PMC8604949; doi:10.1038/s42003-021-02848-x)

Supplementary Figure 1. The density-dependent evaluation of the high-density culture using TkDN-4M hPSC line. **a** The experimental procedure and culture configuration of 4 days expansion period. **b** The aggregates morphology after 4 days expansion of different inoculation density. **c** Diameter measurement of hiPSCs aggregates population. **d** The cross-sectioned hematoxylin-eosin staining of hiPSCs aggregates after 4 days of expansion. **e** The fold expansion and proliferation curve of hiPSCs during expansion. Inoculation density indicated by the black line with one asterisk. The high-density mammalian cell culture is referring to 10^7 cells/ml, shown in blue line with two asterisks. (n= 3 biologically independent experiments). Mean ± standard deviation are indicated in each graph. Statistical significance : ****p<0.0001; ***p<0.001.

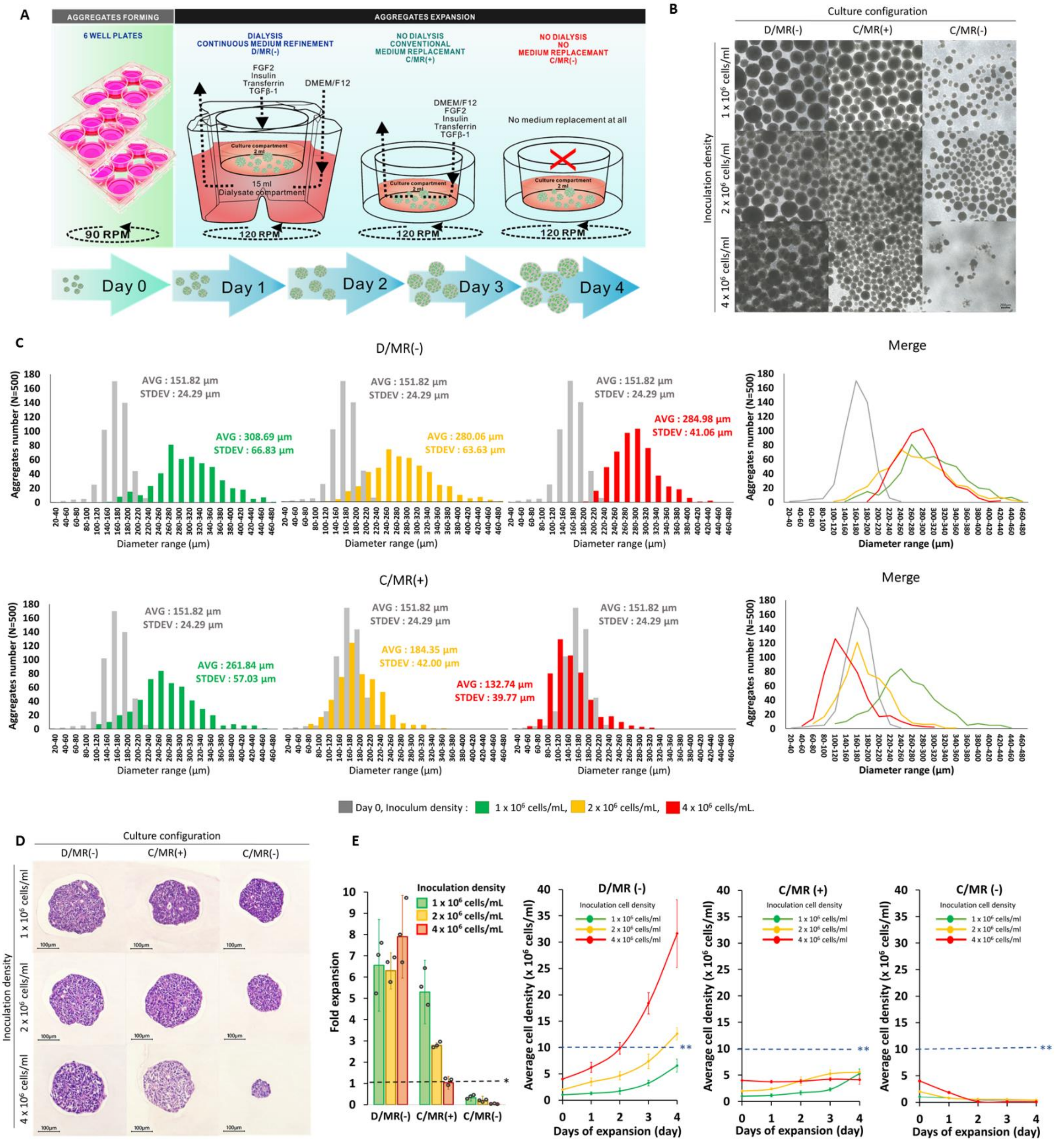

Supplementary Figure 2. Device performance in maintaining the medium refinement in different density of hiPSCs. The concentration of **a** Glucose and **b** Lactate during 4 days of expansion in different inoculation densities using TkDN-4M hPSC line (n= 3 biologically independent experiments). The original glucose concentration is indicated by line with one asterisk. The critical lactate concentration for PSCs culture desribed by Ouyang et al. (2007)[24] indicated in line with two asterisks, and critical lactate concentration for PSCs culture which described by Horiguchi et al. (2018)[23] indicated by line with three asterisks. n=3 biologically independent experiments. Mean ± standard deviation are indicated in each graph.

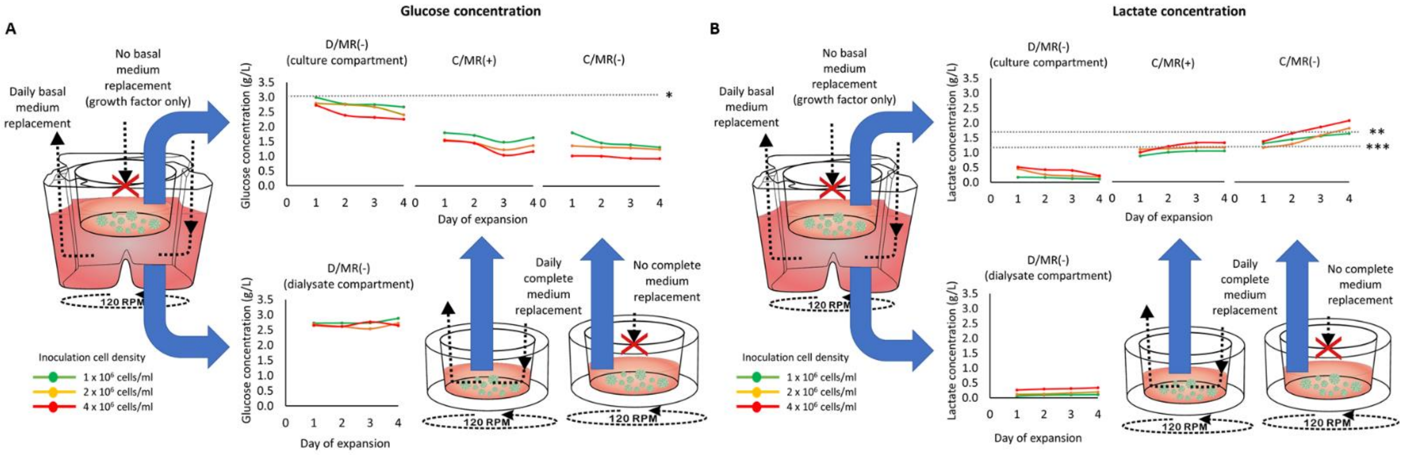

Supplementary Figure 3. Growth factors concentration in different inoculation densities of TkDN-4M hiPSC line during 4 days of hiPSCs expansion. The accumulation of **a** FGF2 and **b** Insulin was detected in D/MR(-), while **c** TGFβ-1 showing a low accumulation. (D). The Nodal concentration was measured at the end of the expansion period. The dotted blue line indicated the daily dose of growth factor in culture medium. Mean ± standard deviation are indicated in each graph (n=3 biologically independent experiments). Statistical significance : ****p<0.0001; ***p<0.001.

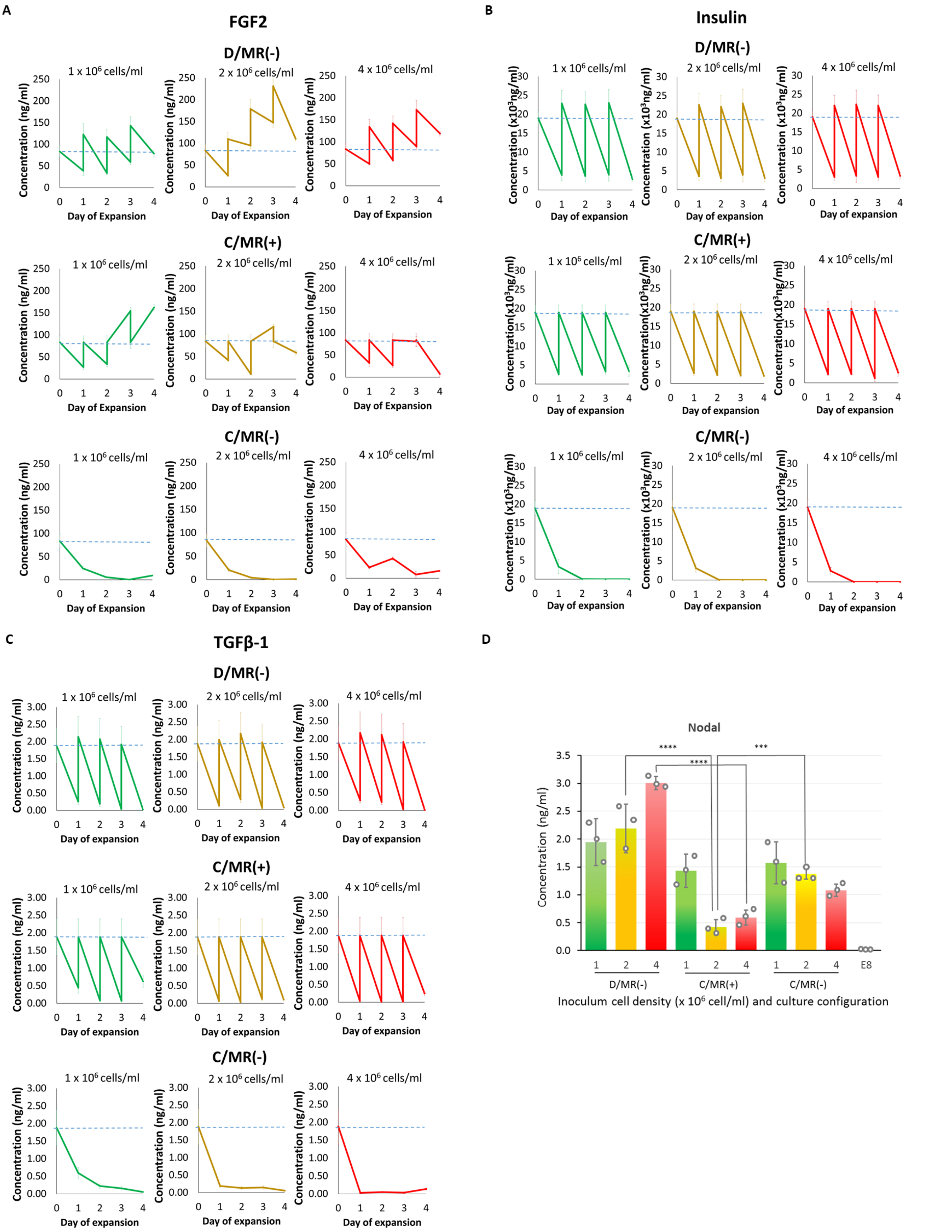

Supplementary Figure 4. The pluripotency gene expression profile in different inoculation cell densities of TkDN-4M hiPSC line after 4 days of expansion (n= 3 biologically independent experiments). Mean ± standard deviation are indicated in each graph (n=3). Statistical significance : ***p<0.001; **p<0.01.

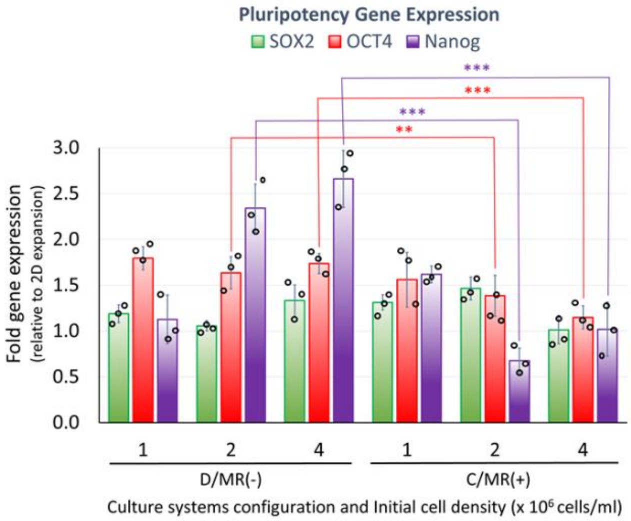

Supplementary Figure 5. The schematic illustration of Nanog regulation by the TGFβ family (such as TGFβ-1 and Nodal) through SMAD2/3 which affecting the balance of the pluripotency by controlling ectoderm and endoderm lineage development.

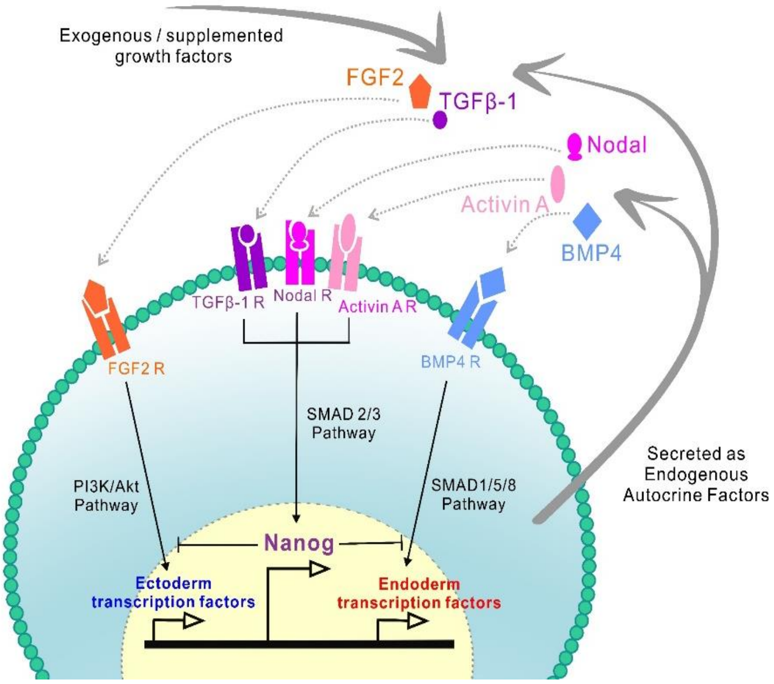

Supplementary Figure 6. (A) The potential cost-saving comparison between the high density expansion in dialysis culture system (D/MR(-)), high density expansion in conventional daily medium replacement (C/MR(+)), and the routine normal density suspension culture, 2.5 x 10^5 cells/ml with daily complete medium replacement (ND) in 5 passages. (B) the percentage of potential cost saving of high-density suspension culture compared to the routine suspension culture in normal density. The calculation was based on exogenous growth factors and basal medium usage and their price in January 2021. (n= calculation based on 3 biologically independent experiments). Mean ± standard deviation are indicated in each graph (n=3). Statistical significance : ****p<0.0001.

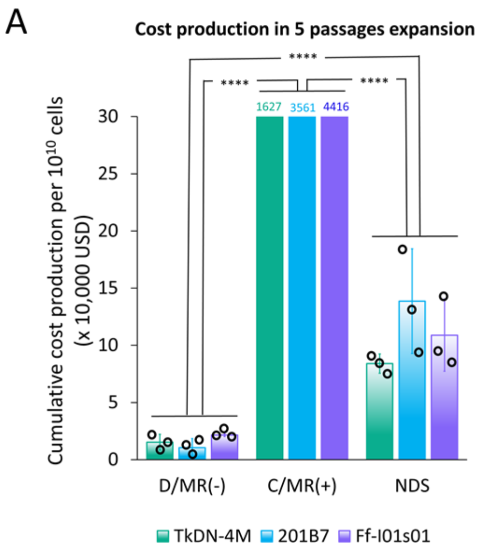

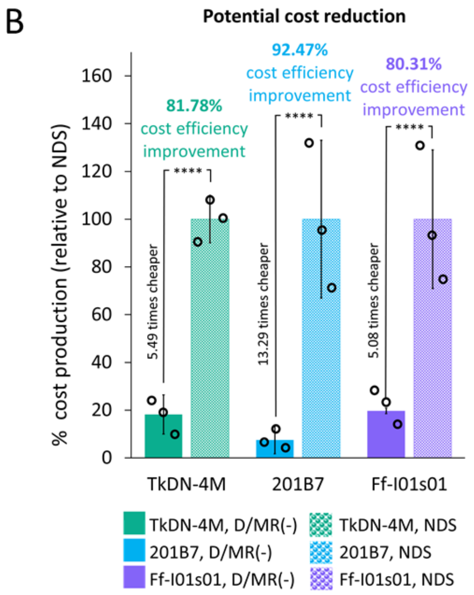

Supplementary Table 1. The list of antibody used in this study

No	Protein target	Antibody / Kit Catalog no.	Application
1	FGF2	Human FGF basic/FGF2/bFGF DuoSet ELISA kit Cat. no. DY233	ELISA
2	Insulin	Human/Canine/Porcine Insulin DuoSet ELISA kit Cat. no. DY8056-05	ELISA
3	TGFβ-1	Human TGFβ-1 DuoSet ELISA kit Cat. no. DY240	ELISA
4	Nodal	Human NODAL ELISA Kit (Sandwich ELISA) Cat. No. LS-F12163	ELISA
5	OCT4, Nanog, SOX2	Human Pluripotent Stem Cell 3-Color Immunocytochemistry Kit, Cat. no. SC021	ICC
6	SSEA4	Alexa Fluor® 488 anti-human SSEA-4 Antibody, Cat. No. 330411	FACS
7	Alkaline Phosphatase (ALP)	Human Alkaline Phosphatase/ALPL Primary Antibody Antibody, Cat no. MAB1448 and Goat Anti-Mouse IgG Alexa Fluor 488 secondary antibody, Cat. No. ab150113	ICC

*validation of each antibody can be found on the manufacturer`s website

Supplementary method 1. Construction of simple miniaturized dialysis culture unit

A. The culture compartment was created by trimming the lower part of a 40-µm mesh bottom-cell strainer (PluriSelect, Leipzig, Germany) until all of the mesh strainers were completely removed.

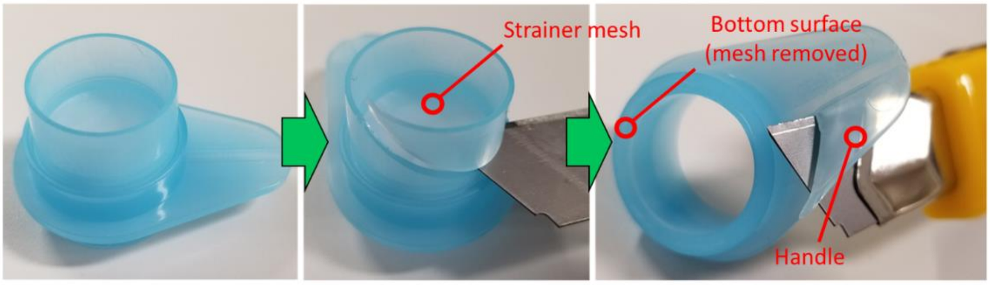

B. To construct the simple dialysis culture unit (D/MR(-)), the Alkyl-α-cyanoacrylate-based surgical-grade tissue adhesive (Aron Alpha A; Daiichi Sankyo, Japan) was uniformly applied to the bottom surface of the culture compartment insert. Afterward, the 12-kDa MWCO Spectra/Por 4 dialysis membrane (Spectrum Chemical, New Brunswick, NJ, USA) was affixed on the bottom part of the insert. The excess dialysis membrane was trimmed. The dialysis culture compartment insert was carefully placed on each deep well of 6-deep well plates (Corning, NY, USA) followed by ethylene oxide gas sterilization.

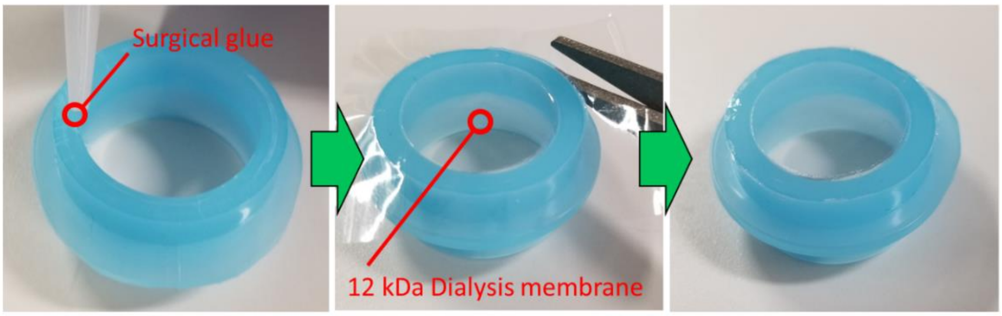

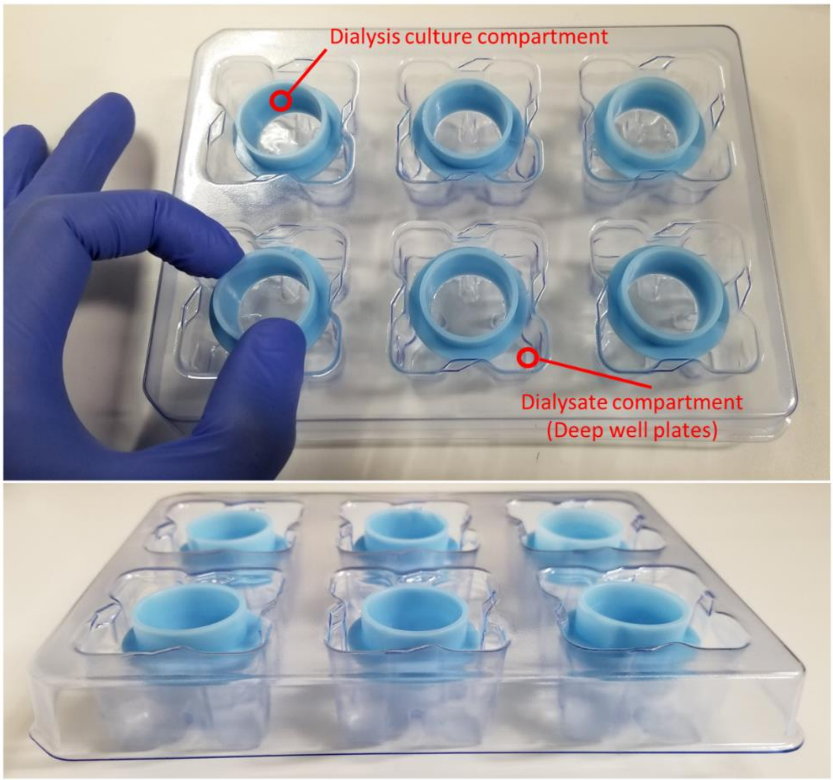

C. As the control with a similar size with the dialysis culture compartment ((C/MR(+) and C/MR(-)), the trimmed strainer then directly affixed in 6 well plates using alkyl-α-cyanoacrylate-based surgical-grade tissue adhesive (Aron Alpha A; Daiichi Sankyo, Japan). The unit was put in a sterilization envelope followed by sterilization using an ethylene oxide gas sterilizer.

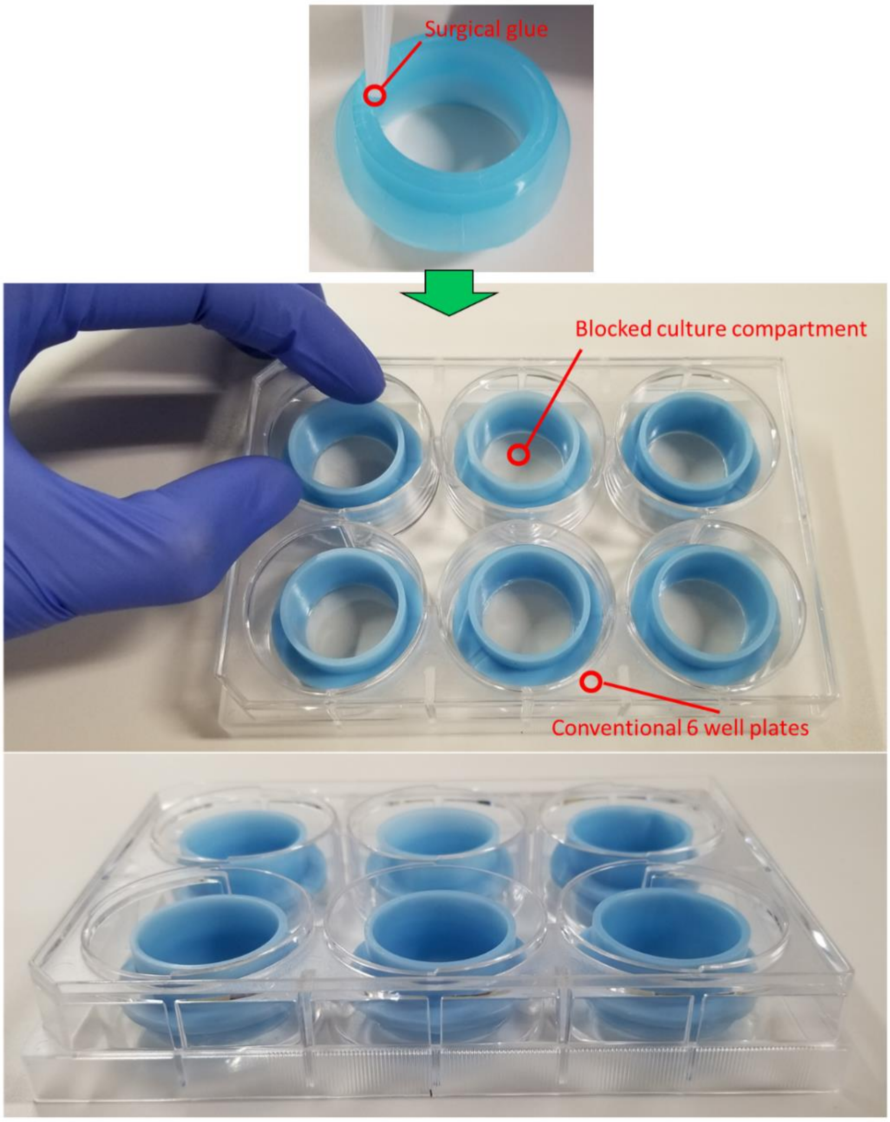

Supplement: Supplementary file 2 — Supplementary Information [file 42003_2021_2848_MOESM2_ESM.pdf]
