# Supplementary material for: A miniature dialysis-culture device allows high-density human-induced pluripotent stem cells expansion from growth factor accumulation
Source: Commun Biol. 2021 Nov 19;4:1316. doi: 10.1038/s42003-021-02848-x (PMC8604949; doi:10.1038/s42003-021-02848-x)
Supplement: Supplementary file 3 — Description of Additional Supplementary Files [file 42003_2021_2848_MOESM3_ESM.pdf]

Description of Additional Supplementary Files

File name: Supplementary Data 1

Description: Source data for graphs and charts.
