# Supplementary material for: A miniature dialysis-culture device allows high-density human-induced pluripotent stem cells expansion from growth factor accumulation
Source: Commun Biol. 2021 Nov 19;4:1316. doi: 10.1038/s42003-021-02848-x (PMC8604949; doi:10.1038/s42003-021-02848-x)
Supplement: Supplementary file 5 — Reporting Summary [file 42003_2021_2848_MOESM5_ESM.pdf]

nature research

Corresponding author(s): Fuad Gandhi Torizal

Last updated by author(s): Oct 8, 2021

Reporting Summary

Nature Research wishes to improve the reproducibility of the work that we publish. This form provides structure for consistency and transparency in reporting. For further information on Nature Research policies, see our Editorial Policies and the Editorial Policy Checklist.

Statistics

For all statistical analyses, confirm that the following items are present in the figure legend, table legend, main text, or Methods section.

n/a	Confirmed	
☐	☒	The exact sample size (*n*) for each experimental group/condition, given as a discrete number and unit of measurement
☐	☒	A statement on whether measurements were taken from distinct samples or whether the same sample was measured repeatedly
☐	☒	The statistical test(s) used AND whether they are one- or two-sided<br>*Only common tests should be described solely by name; describe more complex techniques in the Methods section.*
☐	☒	A description of all covariates tested
☒	☐	A description of any assumptions or corrections, such as tests of normality and adjustment for multiple comparisons
☐	☒	A full description of the statistical parameters including central tendency (e.g. means) or other basic estimates (e.g. regression coefficient) AND variation (e.g. standard deviation) or associated estimates of uncertainty (e.g. confidence intervals)
☐	☒	For null hypothesis testing, the test statistic (e.g. *F*, *t*, *r*) with confidence intervals, effect sizes, degrees of freedom and *P* value noted<br>*Give P values as exact values whenever suitable.*
☒	☐	For Bayesian analysis, information on the choice of priors and Markov chain Monte Carlo settings
☒	☐	For hierarchical and complex designs, identification of the appropriate level for tests and full reporting of outcomes
☒	☐	Estimates of effect sizes (e.g. Cohen's *d*, Pearson's *r*), indicating how they were calculated

Our web collection on statistics for biologists contains articles on many of the points above.

Software and code

Policy information about availability of computer code

Data collection	Fluoview FV-31s-SW for confocal microscopy, StepOne software 2.3 for real time PCR, WinMDI for FACS
Data analysis	GraphPad Prism 8.3.0 for statistical analysis, ImageJ 2.53 for image analysis,

For manuscripts utilizing custom algorithms or software that are central to the research but not yet described in published literature, software must be made available to editors and reviewers. We strongly encourage code deposition in a community repository (e.g. GitHub). See the Nature Research guidelines for submitting code & software for further information.

Data

Policy information about availability of data

All manuscripts must include a data availability statement. This statement should provide the following information, where applicable:

- Accession codes, unique identifiers, or web links for publicly available datasets
- A list of figures that have associated raw data
- A description of any restrictions on data availability

The source data for graphs and charts in this study are available as Supplementary data 1. Further remaining information can be obtained from the corresponding author upon reasonable request.

Field-specific reporting

Life sciences study design

All studies must disclose on these points even when the disclosure is negative.

Sample size	Multiple hiPSC lines were used for each experiment, the biologically independent experiments were repeated at least 3 times for 5 time passages. The number of experiments and standard deviations are reported in the graph and/or figure legends.
Data exclusions	No data was excluded in this study
Replication	We have examined reproducibility in multiple cell lines and experiments. Each analysis involved at least 3 biological replicates
Randomization	Multiple hiPSC lines were expanded in simple dialysis culture system and conventional suspension culture system with at least 3 biological replicates.
Blinding	The blinding was not relevant to this study because the exact similar experimantal group need to be continuously observed and analyzed during expansion period.

Reporting for specific materials, systems and methods

We require information from authors about some types of materials, experimental systems and methods used in many studies. Here, indicate whether each material, system or method listed is relevant to your study. If you are not sure if a list item applies to your research, read the appropriate section before selecting a response.

Materials & experimental systems

n/a	Involved in the study	
☐	☒	Antibodies
☐	☒	Eukaryotic cell lines
☒	☐	Palaeontology and archaeology
☒	☐	Animals and other organisms
☒	☐	Human research participants
☒	☐	Clinical data
☒	☐	Dual use research of concern

Methods

n/a	Involved in the study	
☒	☐	ChIP-seq
☐	☒	Flow cytometry
☒	☐	MRI-based neuroimaging

Antibodies

Antibodies used	All antibodies used are commercially available and widely used, and the catalogue number and/or clone number is reported in supplementary materials.
Validation	All antibody was validated by the manufacturers. Validation data related with species reactivity and application was provided in supplementary material.

Eukaryotic cell lines

Policy information about cell lines

Cell line source(s)	The TkDN-4M hiPSC line was provided by the Stem Cell Bank, Centre for Stem Cell Biology and Regenerative Medicine, University of Tokyo (Tokyo, Japan). The 201B7 and Ff-I01s01 was provided by Center of iPS Research and Applications (CiRA), Kyoto University (Kyoto, Japan)
Authentication	All cell lines were authenticated by providers.
Mycoplasma contamination	The cell line were not tested for mycoplasma contamination
Commonly misidentified lines (See ICLAC register)	No commonly misidentified cell lines were used.

Flow Cytometry

Plots

Confirm that:

☒ The axis labels state the marker and fluorochrome used (e.g. CD4-FITC).

☒ The axis scales are clearly visible. Include numbers along axes only for bottom left plot of group (a 'group' is an analysis of identical markers).

☒ All plots are contour plots with outliers or pseudocolor plots.

☒ A numerical value for number of cells or percentage (with statistics) is provided.

Methodology

Sample preparation	The hiPSC line were expanded at high density in simple dialysis and conventional suspension culture.
Instrument	Coulter EPICS® XL Flow Cytometry System
Software	The WinMDI 2.9 was used for data collection and anaysis
Cell population abundance	All the hPSCs (1 million cells) were collected at the end of expansion peroid followed by dissociation into single cells, stained with and without primary antibodies, and sorted. The purity was determined by SSEA-4 and ALPL staining.
Gating strategy	All samples were preliminarily gated by FSC-A and SSC-A to select singlet cells. The boundary of positive and negative population was determined by FITC-A intensity of cells stained without primary antibody.

☒ Tick this box to confirm that a figure exemplifying the gating strategy is provided in the Supplementary Information.
